# Linked genetic variants on chromosome 10 control ear morphology and body mass among dog breeds

**DOI:** 10.1186/s12864-015-1702-2

**Published:** 2015-06-23

**Authors:** Matthew T. Webster, Nona Kamgari, Michele Perloski, Marc P. Hoeppner, Erik Axelsson, Åke Hedhammar, Gerli Pielberg, Kerstin Lindblad-Toh

**Affiliations:** Science for Life Laboratory, Department of Medical Biochemistry and Microbiology, Uppsala University, Uppsala, Sweden; Broad Institute of MIT and Harvard, Cambridge, MA USA; Bioinformatics Infrastructure for Life Sciences, Department of Medical Biochemistry and Microbiology, Uppsala University, Uppsala, Sweden; Department of Clinical Sciences, Swedish University of Agricultural Sciences, Uppsala, Sweden

**Keywords:** Artificial selection, Dogs, Ear morphology, Body mass, Genome-wide association study

## Abstract

**Background:**

The domestic dog is a rich resource for mapping the genetic components of phenotypic variation due to its unique population history involving strong artificial selection. Genome-wide association studies have revealed a number of chromosomal regions where genetic variation associates with morphological characters that typify dog breeds. A region on chromosome 10 is among those with the highest levels of genetic differentiation between dog breeds and is associated with body mass and ear morphology, a common motif of animal domestication. We characterised variation in this region to uncover haplotype structure and identify candidate functional variants.

**Results:**

We first identified SNPs that strongly associate with body mass and ear type by comparing sequence variation in a 3 Mb region between 19 breeds with a variety of phenotypes. We next genotyped a subset of 123 candidate SNPs in 288 samples from 46 breeds to identify the variants most highly associated with phenotype and infer haplotype structure. A cluster of SNPs that associate strongly with the drop ear phenotype is located within a narrow interval downstream of the gene *MSRB3*, which is involved in human hearing. These SNPs are in strong genetic linkage with another set of variants that correlate with body mass within the gene *HMGA2*, which affects human height. In addition we find evidence that this region has been under selection during dog domestication, and identify a cluster of SNPs within *MSRB3* that are highly differentiated between dogs and wolves.

**Conclusions:**

We characterise genetically linked variants that potentially influence ear type and body mass in dog breeds, both key traits that have been modified by selective breeding that may also be important for domestication. The finding that variants on long haplotypes have effects on more than one trait suggests that genetic linkage can be an important determinant of the phenotypic response to selection in domestic animals.

**Electronic supplementary material:**

The online version of this article (doi:10.1186/s12864-015-1702-2) contains supplementary material, which is available to authorized users.

## Background

The huge phenotypic variation in domestic dog breeds is the result of their unique evolutionary history, which involved two main phases. Firstly, the domestication of dogs from wolves, likely more than 15,000 years ago, involved selection for phenotypes necessary for life with humans [1–3]. Subsequently, in the last few hundred years, a huge variety of breeds were formed from the ancestral dog gene pool, a process that involved extreme population bottlenecks and strong artificial selection. These processes have left impacts on patterns of genetic variation, including long blocks of linkage disequilibrium (LD) [4, 5], increased incidence of deleterious mutations [6, 7] and a high prevalence of inherited disease that varies specific to dog breeds.

Recent studies have identified specific mutations involved in both phases of dog domestication. These studies give insight into the nature and timing of the dog domestication process and selective pressures involved [8, 9]. Scanning the genome for regions of extreme F_ST_ between dogs and wolves and reduced heterozygosity in dogs, consistent with selective sweeps, has identified genetic variants that were likely selected during the descent of modern dogs. These include genes involved in starch digestion and brain function, which may underlie adaptation to new diet and behavioural changes [10, 11]. Studies of genomic variation among dog breeds have also uncovered a catalogue of variants underlying their extreme morphological variation and also potentially behaviour and physiology [12–14]. These include shape of ears, snout and limbs, size, tails, coat type and colour. The simplified genetic basis of normal and pathological inherited traits that segregate within dog breeds coupled with long blocks of LD also makes them an outstanding resource for genetic mapping using genome wide association studies (GWAS). The genetic basis of a large number of traits has been identified by taking advantage of these features [15, 16].

Ear phenotypes are of particular interest because phenotypes of many domestic animals are floppy (drop) compared with their wild ancestors, including cattle, goats, rabbits and pigs. The presence of drop ears as a common correlate of domestication is argued to be related to pleiotropic effects of selection for tameness [17, 18]. In support of this, selective breeding experiments to produce tame foxes also resulted in the emergence of drop ears and a suite of other peadomorphic characteristics [19]. Present day dog breeds show huge variation in ear morphology, from pricked ears seen in German Shepherds to large hanging drop ears of Basset Hounds. Ear morphology is included in breed standards and has clearly been under strong artificial selection for these various divergent types.

A region on CFA10 (9.8 - 11.8 Mb canFam2.0) harbours a locus with highly divergent SNP frequencies between dog breeds. F_ST_ values in this region are among the highest in the genome [12–14]. Across-breed GWAS identified a strong association with ear type in this region. There is also a weaker association with body mass. The strongest correlation with size between dog breeds has been found to be on CFA15, close to the *IGF1* gene [20] and the region on CFA10 is secondary to this [12, 13]. Derived variants at six loci, including these two have been shown to account for 64.3 % of variance in weight among breeds with standard weights (<41 kg) [21]. Whereas a single locus in the CFA10 region correlates strongly with the ear phenotype of almost all drop and prick ear breeds [12, 13], body mass correlates with a variant at high frequency in a subset of small breeds [12, 21]. The strongest associations with both body mass and ear type identified from GWAS lie in a region 3′ (downstream) of the methionine sulfoxide reductase (*MSRB3*) gene and 5′ (upstream) of the high-mobility group AT-hook 2 (*HMGA2*) gene [12]. Interestingly the region also shows weaker correlation with boldness [12]. However the biological relevance of this correlation is difficult to evaluate, as it is based on subjective phenotype, which showed a strong covariation with ear type in previous analysis [12]. It is clear however that patterns of variation in this region indicate that it is a key region controlling variation in morphology among dog breeds, and potentially also important for mediating phenotypic changes that occurred during dog domestication.

Here we identified variants that are potential candidates for controlling phenotypic variation in ear morphology and body mass within this region on canine chromosome 10. We first performed targeted sequencing of the region in five pools of samples, each from a single dog breed, that differ in ear morphology and body mass. We compared patterns of variation in these sequences with those obtained from whole genome resequencing of a further five pools of dogs from various breeds and one pool of wolves. This enabled us to identify candidate SNPs that we then genotyped in a larger panel of dogs from various breeds, which allowed inference of haplotypes with strongest correlation to phenotype. We also analysed patterns of variation in this region in dogs compared with wolves and show that it has evidence for selection during dog domestication and identified a cluster of wolf-dog fixations, which could represent SNPs under selection during domestication.

## Results

### Across-breed GWAS identifies interval on chromosome 10

**Fig. 1:**
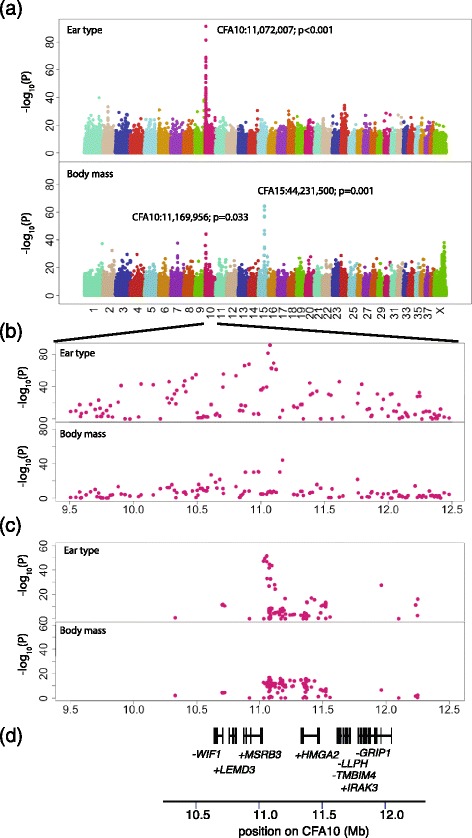
Genetic associations with ear type and body mass among dog breeds. **a** Manhattan plot showing raw p-value of association with ear type (upper panel) and body mass (lower panel) among dog breeds across ~174,000 SNPs. The most significant associations with ear type are found within a region 9.5–12.5 Mb on CFA10. The most significant association with body mass is found on CFA15, close to the *IGF1* gene. The CFA10 region associated with ear type is the second most strongly associated region for body mass. **b** Expanded view of the CFA10 region showing association with ear type (upper panel) and body mass (lower panel). **c** Significance of association between allele frequency and ear type (upper panel) and body mass (lower panel) at 123 candidate SNPs within a ~2 Mb region on CFA10 in 288 samples from 46 breeds. **d** Position of human RefSeq genes mapped onto the canFam2.0 reference. Genes are labelled +/− according to direction of transcription

We first tested for associations with body mass and ear type using a set of 509 samples from 46 breeds (Table 1) typed using the canineHD SNP array (~174,000 SNPs). We performed a GWAS for ear type comparing 20 drop ear breeds (*n* = 242), 12 prick ear breeds (*n* = 108) and 14 intermediate ear breeds (*n* = 159). We estimated genome-wide significance using the breed permutation procedure used in ref. [12]. This method accurately determines significance correcting for different sample sizes of each breed. In total, 24 SNPs reach genome-wide significance. Of these, 23 are found between 9.9 and 11.8 Mb on CFA10 (Fig. 1a,b), with the other on CFA1 (Additional file 1: Table S1). The SNP with the strongest association with ear type is located at CFA10:11,072,007 (p_raw_ = 7.5 × 10^−92^, p_genome-wide_ < 0.001), which lies between the *MSRB3* and *HMGA2* genes (all coordinates given on canFam2.0 assembly).

**Table 1:** Samples used in GWAS with ear and body mass phenotypes

Breed	N	Ear type	Body mass (kg)
Cavalier King Charles Spaniel	5	drop	6
Dachshund	12	drop	7
Beagle	10	drop	10
Cocker Spaniel	14	drop	13
English Cocker Spaniel	2	drop	14
Brittany Spaniel	12	drop	17
Nova Scotia Duck Tolling Retriever	23	drop	20
English Springer Spaniel	3	drop	23
Shar Pei	11	drop	24
Dalmatian	7	drop	25
Standard Poodle	12	drop	25
Weimaraner	26	drop	28
Flatcoated Retriever	2	drop	29
Large Munsterlander	1	drop	30
Labrador Retriever	14	drop	30
English Setter	12	drop	31
Gordon Setter	25	drop	31
Golden Retriever	14	drop	32
Bernese Mountain Dog	12	drop	45
Newfoundland	25	drop	64
Yorkshire Terrier	12	intermediate	3
Border Terrier	25	intermediate	6
Jack Russell Terrier	12	intermediate	7
Pug	2	intermediate	7
Border Collie	16	intermediate	17
Schnauzer	3	intermediate	17
English Bulldog	13	intermediate	24
Australian Shepherd	1	intermediate	25
English Bull Terrier	8	intermediate	25
Boxer	8	intermediate	29
Greyhound	11	intermediate	30
Doberman Pinscher	25	intermediate	35
Rottweiler	12	intermediate	45
Irish Wolfhound	11	intermediate	54
Chihuahua	2	prick	2
Schipperke	25	prick	7
Finnish Spitz	12	prick	11
Czechoslovakian Wolf Dog	3	prick	23
Elkhound	12	prick	23
Eurasier	12	prick	24
Siberian Husky	2	prick	24
Samoyed	2	prick	24
Greenland Sledge Dog	12	prick	31
Belgian Tervuren	12	prick	32
German Shepherd	12	prick	37
Sarloos	2	prick	37
TOTAL	509

We next examined the association with body mass, measured in kilograms, using average mass for each breed (Table 1) using a quantitative association study of all 46 breeds. We identified 8 SNPs with genome wide significance on CFA15 within a narrow region 44.22 - 44.28 Mb. The most associated SNP is at CFA15:44,231,500 (p_raw_ = 4.3 × 10^−65^, p_genome-wide_ = 0.001). These SNPs overlap the *IGF1* locus previously implicated in body mass variation among dog breeds [20]. However, a secondary peak is observed within the region on CFA10 also associated with ear type. One SNP in this region reaches genome wide significance (CFA10:11,169,956, p_raw_ = 8.2 × 10^−45^, p_genome wide_ = 0.033), which lies between *MSRB3* and *HMGA2* (Fig. 1a,b, Additional file 1: Table S1).

There is no significant difference in average body mass between breeds of different ear types in our dataset (Kruskal-Wallis chi-squared = 0.224, *p* = 0.89). The average body mass of drop ear, prick ear and intermediate ear breeds is 25.2 kg, 22.9 kg and 23.1 kg respectively. This indicates that the associations between body mass and ear type in the CFA10 region are independent of each other. We also performed GWAS for body mass within each of the three categories of ear type (drop, prick, intermediate). Among 12 prick ear breeds there was a strong genome wide significant association with body mass on CFA15 near the *IGF1* gene (44,231,500, 44,267,011, 44,226,659, p_genome-wide_ < 0.001) but the signals within the CFA10 region were abolished, without any suggestive signals (Additional file 2: Figure S1). Among the 20 drop ear breeds, there was no significant association anywhere in the genome including the CFA15 and CFA10 regions. However, among 14 breeds with variable or intermediate ear types, the strongest signal was seen in the CFA10 region, with the highest significance near a SNP identified previously using all breeds (CFA10:11,169,556; p_genome-wide_ = 0.097; Additional file 2: Figure S1, Additional file 1: Table S1). These results confirm that the genetic association with body mass is independent of ear type. The lack of association with the CFA10 region in prick and drop ear breeds is likely influenced by the low number of very small breeds with either prick or drop ears in this dataset (Table 1).

In addition to correlations with morphology, previous studies have identified this CFA10 region as being one of the most highly differentiated among breeds [12, 13]. In the same dataset of 46 breeds, a region of 2.0 Mb (CFA10:9.8 - 11.8 Mb) contains 33 SNPs with F_ST_ > 0.55 and minor allele frequency >15 %, representing the second-longest such stretch of SNPs with high F_ST_ in the genome. The SNPs with highest F_ST_ in this region are CFA10:11,169,956 (F_ST_ = 0.81), which is highly associated with body mass and CFA10:11,000,274 (F_ST_ = 0.77) with is highly associated with ear type (see above). The extreme population differentiation in this region is indicative of strong artificial selection.

### Analysis of sequence variation in 3 Mb encompassing the critical interval

The evidence above suggests that a critical region on CFA10 harbours genetic variants responsible for ear type and body mass and has experienced selection due to the creation and maintenance of different dog breeds. We therefore decided to assay sequence variation in a 3 Mb interval encompassing this region (CFA10: 9.5 Mb - 12.5 Mb) in breeds with a variety of phenotypes in order to identify candidate genetic variants that control this variation. This region was selected to encompass the 1–2 Mb highly differentiated interval defined by *F*_*ST*_ identified by refs [12, 13]. Using sequence capture followed by sequencing of a lane of Illumina Hi-Seq per library, we sequenced this interval in 5 pools of dogs each containing 5 samples from the same breed, resulting in average coverage of 4,227x. We chose breeds with either drop or non-drop ears that were fixed for the appropriate alleles at associated SNPs in the GWAS analyses and the segregation of associated markers presented in ref. [21]. These consisted of two small breeds with non-drop ears (Border Terrier, Jack Russell Terrier), one large breed with non-drop ears (German Shepherd), and two large breeds with drop ears (Weimeraner, English Springer Spaniel; Table 2). The two small breeds are expected to harbour the small mass variant according to the results from GWAS presented above and the segregation of associated markers presented in ref. [21]. We refer to this as the sequence capture (SC) dataset.

**Table 2:** Samples used in resequencing studies with numbers of SNPs identified using stringent cutoff (99 %)

Code	Breeds	N	Body mass	Ear	Depth (x)	Fixed ref allele	Polymorphic	Fixed non-ref allele	Uninformative
*SC pools*									
BT	Border Terrier	5	small	non-drop	4,748	2,359	1,092	1,630	100
JR	Jack Russell Terrier	5	small	non-drop	4,910	966	3,562	596	57
GS	German Shepherd	5	large	non-drop	5,182	1,442	2,489	1,165	85
WEI	Weimeraner	5	large	drop	4,305	1,648	2,923	513	97
ESS	English Springer Spaniel	5	large	drop	1,990	2,160	2,296	531	194
*WGS pools*									
Pool 1	Wolf	12	large	non-drop	7.5	2,450	1,397	449	885
Pool 2	Smaland Hound, Norwegian Elkhound, Swedish Elkhound, Finnish Lapphund	12^a^	large	mix	6.9	2,135	1,688	405	953
Pool 3	Cocker spaniel, Springer Spaniel, Golden Retriever, Labrador Retriever	12^a^	large	drop	6.1	2,426	1,391	300	1,064
Pool 4	Drever	12	large	drop	8.0	2,891	862	606	822
Pool 5	Belgian Tervuren	12	large	non-drop	8.1	2,281	1,508	531	861
Pool 6	Bearded Collie, Hovawart, Riesenschnauzer, German Shepherd	12^a^	large	mix	7.9	2,046	2,172	187	776

^a^3 of each breed

**Fig. 2:**
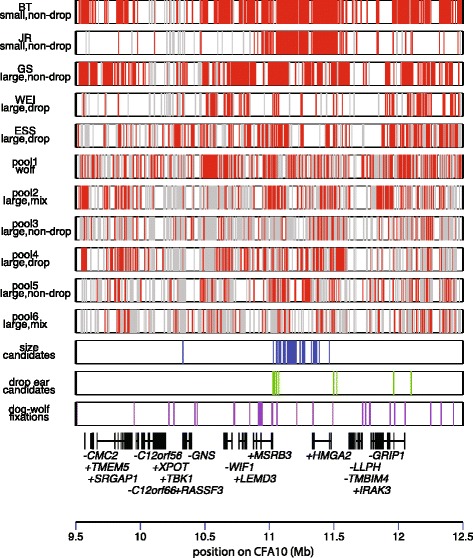
Patterns of SNP variation in a 3 Mb region on CFA10. The first 5 bars show variation in the sequence capture (SC) pools of single breeds and the next 6 bars show variation in the whole genome sequencing pools (WGS; see Table 2 for details). Red lines represent SNP positions that are fixed for a non-reference allele in a particular pool, grey lines represent SNP positions that cannot be confidently assessed due to low coverage. Sites that are polymorphic within a breed, or that match the reference allele are not marked. The bottom 3 bars represent SNPs that display patterns of fixation that matches phenotypic variation. Candidate SNPs for controlling variation in body mass (blue) ear type (green) and those that are fixed for alternate alleles in all dogs compared to wolves (purple) are shown. The location of protein coding genes in the region are also shown, which were identified by mapping human RefSeq genes onto the canFam2.0 dog assembly. Genes are labelled +/− according to direction of transcription. Ear and body mass candidates are concentrated in a region between the *MSRB3* and *HMGA2* genes, whereas a cluster of dog-wolf fixations is found within the *MSRB3* gene

We identified common SNPs in the SC dataset based on a cutoff of minor allele frequency >0.1 and then inferred the frequency of each SNP in each pool in all samples based on proportion of reads matching each allele (see methods). We identified 5,181 variable SNPs in the SC data using this approach. Each SNP was then classified as fixed for reference allele, fixed for non-reference allele, polymorphic or uninformative in each pool (Fig. 2, Table 2, Additional file 3: Table S2) using a selection of both stringent and loose cutoffs to define fixation. From among these SNPs we identified candidates that segregated with the body mass or ear phenotypes. In order for a SNP to be considered a candidate, it was necessary for all pools representing a particular phenotype to be fixed for the same allele. In total, 83 ear type candidates and 87 body mass candidates were identified from the SC data.

We next compared patterns of variation in SNPs identified in the sequence capture sequences with reads from whole genome sequencing (WGS) mapped to the same region in 5 pools of dog samples from one or more breeds, and one pool of wolf samples presented by Axelsson *et al.* [10]. All of the dog pools comprised large breeds. Two of these dog pools contained only drop ear breeds, one contained a single prick ear breed and two contained breeds with a mixture of ear types (Table 2). The wolf pool was considered as having a large body mass and prick ear phenotype. Only positions that were variable in the sequence capture pools were considered in the WGS pools, which were also defined as fixed for reference allele, fixed for non-reference allele, polymorphic or uninformative.

We utilized patterns of segregation in the WGS pools to rule out candidate SNPs from the SC pools that showed patterns of segregation inconsistent with phenotype. SNP candidates were filtered if alleles matching the incorrect phenotype based on the SC data were observed in any WGS pool (see Additional file 3: Table S2 for full set of SNPs). The remaining candidate SNPs are mainly concentrated in a 500 kb region between 11.0 and 11.5 Mb, which is downstream of the *MSRB3* gene and encompasses the *HMGA2* gene. A cluster of seven candidate SNPs for ear type are found immediately downstream of the *MSRB3* gene between 11.0 and 11.1 Mb (Fig. 2).

We performed an analysis based on read depth in order to identify putative copy number variants (CNVs) that associated with phenotype but did not identify any such cases. We used the SC data to scan the 500 kb associated region and flanking sequence using 100 bp windows to identify asymmetrical read depth between pools that could result from copy number variation (Additional file 4: Figure S2). We inspected the pileup of reads around 28 regions with more than twofold variation in read depth or where one or more pool had no coverage using integrative genome viewer (IGV). Of these, 22 regions mapped to repetitive elements, including two that mapped to simple repeats and 20 that mapped to LINE/SINE elements (Additional file 5: Table S3). Although some of these may represent true CNVs related to presence/absence of repeat elements, the patterns are consistent with poorly mapped reads. Out of all the regions only 5 have some degree of conservation and none of these show patterns of relative read coverage consistent with a correlation to either the ear or body mass phenotypes. There are therefore no strong candidates among these regions that may indicate structural variation governing phenotype.

### Genotyping of candidate SNPs identifies haplotypes associated with both traits

We selected 123 SNPs for further genotyping including all of the candidate SNPs identified using the strict criteria presented above, augmented with additional SNPs that were candidates at lower thresholds for fixation. All candidates for ear and body mass were included from the strict dataset and in total we genotyped 83 body mass candidates and 40 ear candidates (marked in Additional file 3: Table S2). We genotyped 288 samples from 46 breeds including 11 with prick ears, 18 with intermediate ears and 17 with drop ears (Table 3) and analysed association between allele frequencies and phenotype (Additional file 6: Table S4). Figure 1c shows significance of correlations between body mass and ear type across all SNPs (see also Additional file 7: Table S5 for full results).

**Table 3:** Haplotypes identified in genotyped breeds

				Haplotype						
Breed	Samples	Ear^a^	Body mass (kg)	D	L1	L2	L3	S1	S2	Other
Chinese Crested	8	1	5	0	0	1	0	8	1	6
Schipperke	8	1	7	0	1	0	0	12	0	1
Basenji	8	1	11	0	0	1	0	0	15	0
Finnish Spitz	8	1	11	0	10	0	0	0	0	6
Pembrokeshire Welsh Corgi	7	1	12	2	8	0	0	2	0	0
Border Collie	8	1	17	0	16	0	0	0	0	0
Norwegian Elkhound	5	1	23	0	4	0	0	0	6	0
Chow Chow	2	1	25	0	3	0	0	0	0	1
Samoyed	4	1	25	0	8	0	0	0	0	0
German Shepherd	5	1	31	0	10	0	0	0	0	0
Akita	3	1	55	0	3	3	0	0	0	0
Border Terrier	10	2	6	0	18	0	0	0	0	2
Minature Schnauzer	5	2	6	0	10	0	0	0	0	0
Fox Terrier	5	2	7	0	0	0	0	5	0	5
Jack Russell	2	2	7	0	0	0	0	4	0	0
Pug	5	2	7	0	0	1	0	8	0	1
Australian Shepherd	5	2	22	1	3	0	1	0	0	1
Airedale Terrier	5	2	24	0	10	0	0	0	0	0
Boxer	5	2	29	0	2	8	0	0	0	0
Greyhound	5	2	33	0	8	0	0	0	0	0
American Staffordshire Terrier	5	2	34	3	4	0	3	0	0	0
Doberman	4	2	35	1	6	0	1	0	0	0
Giant Schnauzer	5	2	38	4	4	0	2	0	0	0
Rottweiler	5	2	49	1	8	0	1	0	0	0
Irish Wolfhound	5	2	55	0	10	0	0	0	0	0
Pappillon	7	2^b^	4	0	8	2	0	4	0	0
Phalène	10	2^b^	4	1	8	2	0	5	0	4
Norfolk Terrier	10	2^b^	5	0	0	0	0	20	0	0
Norwich Terrier	10	2^b^	5	0	0	0	0	19	0	1
Cavalier King Charles Spaniel	7	3	6	0	0	0	0	0	0	2
Havanese	4	3	6	3	1	0	0	0	0	4
Dachshund	10	3	7	13	7	0	0	0	0	0
Beagle	2	3	10	3	0	0	0	1	0	0
Tibetan Terrier	3	3	11	0	0	0	0	6	0	0
Cocker Spaniel	3	3	13	6	0	0	0	0	0	0
Lagotto Romagnolo	8	3	14	13	1	0	0	0	0	2
Poodle	8	3	25	16	0	0	0	0	0	0
Basset Hound	8	3	28	16	0	0	0	0	0	0
Irish Setter	8	3	28	16	0	0	0	0	0	0
Golden Retriever	4	3	30	6	0	0	2	0	0	0
English Setter	8	3	31	13	0	0	0	0	0	1
Bernese Mountain Dog	8	3	45	15	1	0	0	0	0	0
Leonberger	7	3	60	14	0	0	0	0	0	0
Newfoundland	9	3	64	11	0	0	0	0	0	3
Great Dane	10	3	70	16	3	0	0	0	0	1
Saint Bernhard	7	3	90	6	0	5	0	0	0	1
TOTAL	288			180	175	23	10	94	22	42

^a^1 = prick, 2 = intermediate, 3 = drop

^b^Phalène is a breed closely related to Pappillon with comparatively more dropped ears. Norfolk terrier is drop ear breed closely related to Norwich terrier with comparatively more dropped ears

We identified seven SNPs within a ~60 kb window at CFA10:11.02-11.08 Mb, which lies immediately 3′ of the gene *MSRB3*, that are strongly associated with ear type. A larger number of SNPs showed associations with body mass across a large (~400 kb) interval (CFA10:11.02-11.43 Mb) that encompasses the ear type region and extends into the 5′ end of the gene *HMGA2* (Figure 1c). The associations with body mass are weaker but extend across a much larger region. The presence of multiple SNPs with similar levels of association across this region is indicative of them being in LD. This additional genotyping therefore enables us to further filter the list of candidate variants from the resequencing study, and identifies multiple genetic variants associated with ear type and body mass within a reduced interval.

We next repeated the associations with body mass in subsets of data divided according to ear type (Additional file 8: Figure S3). Consistent with the previous equivalent GWAS analysis (Additional file 2: Figure S1) the strongest associations are observed within the 18 intermediate ear breeds, with associations shown across the same set of SNPs as observed across all breeds. Weaker associations with body mass are identified among 11 prick ear breeds, whereas there are no notable associations with body mass in this region among drop ear breeds although the latter result is likely due to the low number of small drop ear breeds in the dataset (Additional file 8: Figure S3). These results confirm that variation within this region correlates with body mass independently of ear type, suggesting that these two phenotypes are controlled by separate genetic variants within the region.

**Figure 3:**
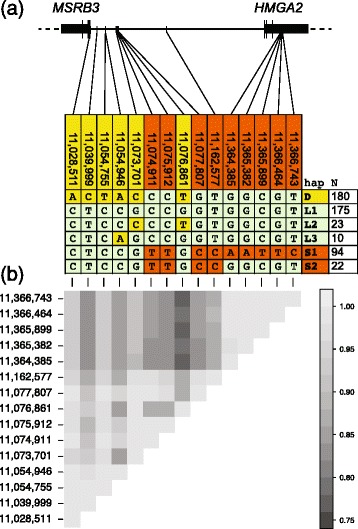
Haplotype structure inferred across 15 SNPs highly associated with ear type or body mass and patterns of linkage disequilibrium. **a** Locations of SNPs on the haplotype relative to the *MSRB3* and *HMGA2* genes. SNPs and haplotypes associated with ear type are highlighted yellow whereas those associated only with body mass are highlighted orange. Only haplotypes present >7 times in the dataset are shown. **b** Pairwise estimates of linkage disequilibrium measured by |D’|

We selected 15 SNPs with the strongest associations to ear type (raw *p* < 10^−45^) and/or body mass (raw *p* < 10^−15^) spanning 340 kb and inferred haplotypes present in each sample at these SNPs. We were able to infer the haplotypes present in 273 of 288 samples. In total, we inferred 29 different haplotypes. The six haplotypes that are present at frequencies >1.5 % in the dataset are shown in Fig. 3a and the occurrence of these haplotypes in each breed is shown in Table 3 (data for all haplotypes are presented in Additional file 9: Table S6). Drop ear breeds predominantly carry haplotype D, which occurs very rarely in other breeds (Table 4). This haplotype carries the minor allele for a cluster of ear-type-associated SNPs in a 5′ portion of the interval. Haplotypes S1 and S2 occur predominantly in small breeds without drop ears, and are rare in other breeds. These haplotypes carry the minor alleles for a cluster of body-mass-associated SNPs in the 3′ portion of the interval. Haplotypes L1 and L2 are most common in larger breeds without drop ears, but are also present in other breeds.

**Table 4:** Distribution of haplotypes among breeds

	Haplotype							
Breed phenotype	D	L1	L2	L3	S1	S2	Other	Total
Drop ear, <15 kg	38	9	0	0	7	0	8	62
Non-drop-ear, <15 kg	3	63	7	0	87	16	26	202
Drop ear, >15 kg	129	4	5	2	0	0	6	138
Non-drop-ear, >15 kg	10	99	11	8	0	6	2	144
Total	180	175	23	10	94	22	42	546

These observations suggest that the D haplotype harbours one or more variants that cause drop ears whereas the S haplotypes harbour one or more variants that cause low body mass. The association between body mass and haplotype variation in this region is weaker than with ear type, which is likely due to the presence of additional modifiers elsewhere in the genome, most notably at the *IGF1* locus [20, 21]. Recombinant haplotypes that carry subsets of the SNPs associated with ear type and body mass are observed, although extremely rarely (<1 %) and homozygotes are not observed. Breeds that have both drop ears and low body mass harbour a mixture of D and S haplotypes (Table 4, Additional file 9: Table S6) suggesting that this phenotype is not caused by fixation of a haplotype possessing both the drop ear and low body mass variants.

We analysed pairwise LD between the 15 associated SNPs using both |D’| (Fig. 3b) and r^2^ (Additional file 10: Figure S4). These analyses reveal two blocks of near-perfect LD corresponding to the 5′ and 3′ SNP clusters that associate with ear type and body mass respectively. Within these clusters, mean |D’| between SNPs is 0.96 and mean r^2^ is 0.88. The two blocks are also in strong LD with each other measured by |D’| (mean |D’| between SNPs from different blocks is 0.88). This reflects the apparent lack of recombinant haplotypes in the region (Fig. 3b). However, correlation between SNPs within these two haplotype blocks measured by r^2^ is lower (mean r^2^ = 0.23; Additional file 10: Fig. S4) which reflects the observations that there are three major haplotypes and that the alleles associated with ear type and body mass are rarely found on the same haplotype and therefore not strongly correlated.

The breeds we genotyped included two pairs of breeds that are known to be closely related but vary to some extent in ear type. The Norwich Terrier has more pricked ears than the closely related Norfolk Terrier. These two breeds were considered to be the same breed by kennel clubs until the 1960–1970s. The Papillon has more pricked ears compared to the Phalène breed, and the two forms may appear in the same litter. There was however clearly no differentiation of this region between these pairs of breeds and the most ear-associated SNPs were homozygous for the prick ear type in all four of these breeds (Additional file 6: Table S4). The Norwich and Norfolk Terriers both predominantly possess the S1 haplotype whereas the Phalène and Pappillon both possess a mixture of L1 and S1 haplotypes (Table 3). It is therefore highly unlikely that genetic variation in this region controls differences in ear type between these specific breeds.

### Comparison with genetic variation in wolves reveals putative signals of selection

**Fig. 4:**
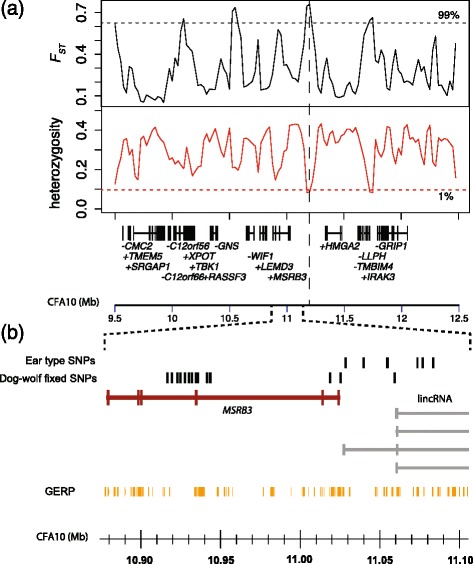
Patterns of genetic variation and candidate SNPs. **a** Variation in heterozygosity within dogs and F_ST_ between wolves and dogs in a 3 Mb region on CFA10 encompassing the critical interval associated with ears and body mass. Both statistics were measured in 40 kb windows. Horizontal dotted lines represent cutoff values for percentiles across the entire genome. A region with extremely high F_ST_ and extremely low heterozygosity (11.15–11–-25 Mb) is marked by a vertical dotted line. **b** Detailed view of the SNPs most associated with ear type, which are clustered downstream of the *MSRB3* gene and the SNPs that are fixed for alternate alleles between wolves and dogs, including a cluster of SNPs within the *MSRB3* gene. The ear type associated SNPs are located at sites that map to lincRNA transcripts in the human genome, whereas the cluster of dog-wolf fixed SNPs are found in introns of *MSRB3*. Also shown are the GERP conserved elements derived from a 39 eutherian mammal alignment [50]

We next analysed the CFA10 region for signatures of selective sweeps. We estimated levels of heterozygosity in dogs and F_ST_ between dogs and wolves across the genome in 40 kb windows (Fig. 4a). One region downstream of *MSRB3* and upstream of *HMGA2* exhibits heterozygosity below the 1 % percentile and F_ST_ above the 99 % percentile compared to 40 kb windows in the entire dog genome (11.15–11-25 Mb), which is potentially indicative of a selective sweep. The region 11.0–11.1 Mb shows very high heterozygosity which is consistent with the presence of two haplotypes corresponding to the drop and prick eared phenotypes in this region. We leveraged the data from both sequence capture and WGS sequences to identify genetic variants that were fixed in dogs and wolves in this region (Fig. 2, Additional file 11: Table S7). We identified 45 such variants within the 3 Mb sequenced region, of which 12 are clustered within 26.7 kb region at CFA10:10,916,652 - 10,943,326 within the *MSRB3* gene. The density of SNPs within this region is 2.2 kb/SNP whereas in the rest of the region it is 130.5 kb/SNP (Fisher’s exact test *p* < 2.2e^−16^). These SNPs are close to the cluster of SNPs that most strongly correlate with ear type (Fig. 4b).

### Functional candidates

We have identified sets of SNPs strongly associated with ear type and body mass respectively and another set which have highly differentiated allele frequencies between dogs and wolves. Figure 4b shows the location of seven ear-type-associated and 12 dog-wolf differentiated SNPs in the vicinity of the *MSRB3* gene. The ear type associated SNPs in the region CFA10:11.02–11.08 Mb are immediately downstream of the *MSRB3* gene (CDS: 10.88–11.02 Mb) and 260 kb upstream of *HMGA2* (CDS: 11.34–11.48 Mb).

MSRB3-catalyzed reduction of methionine sulfoxides to methionine is essential for hearing [22] and a non-synonymous substitution in this gene causes deafness and expression of *MSRB3* in the inner ear is localized in the auditory and vestibular sensory epithelia. There is therefore evidence that *MSRB3* may be involved in ear function, and the SNPs could potentially exert their functional effects on ear morphology by modifying its expression, although a putative mechanism is elusive. We do not identify overlap between any of the SNPs and evolutionary constrained elements. Likewise, none of the SNPs are located within a known coding-region. Interestingly, although previous RNA-seq experiments across multiple tissues have not identified transcription in this region [23], all seven SNPs lie within the coordinates of human lincRNA candidates mapped into dog. These variants could therefore be involved in regulation of gene expression by lincRNA, and could potentially affect expression of *MSRB3* or *HMGA2*. The cluster of dog-wolf fixed SNPs within the *MSRB3* gene is restricted to intronic regions and the SNPs show no overlap with conserved elements or coding nucleotides. Any functional consequences of these SNPS are most likely to be regulatory.

SNPs across the entire 340 kb haplotype region show similar levels of association with body mass. These include a cluster of SNPs within an intron of the *HMGA2* gene, which is a strong candidate for involvement in body mass variation and correlates with several morphological phenotypes including height in humans [24, 25]. One of these SNPs, CFA10:11,364,385, is found within a conserved element and is a good candidate for influencing body mass by affecting expression of *HMGA2*. In the human genome, this SNP maps to a position (chr12:66,247,497) overlapping a H3K27Ac mark in HUVEC cells, suggesting a function in endothelial cells. It is also found within a DNaseI hypersensitive cluster observed in several cell types and an RNA polymerase II transcription factor binding site assayed by ChIP-seq [26] in multiple cell lines suggesting that it affects transcription.

## Discussion

The genetic basis of phenotypic variation is simplified in dogs due to inbreeding and strong artificial selection for variants with large effect [12, 13]. One of the challenges of identifying genetic variants for certain traits is that they may be correlated with each other as regions with high divergence between breeds often show correlations with many phenotypes, some of which may be spurious due to co-occurrence of phenotypes either by chance or due to selection for specific combinations of traits. Here we confirm that a previously identified region on canine chromosome 10 associates with differences in ear type and body mass between dog breeds. Our detailed characterisation of this region indicates that it harbours at least two distinct genetic variants that independently influence these traits.

Our analysis suggests that genetic variants controlling two traits are found on haplotypes that span a 340 kb region on chromosome 10 encompassing a region 3′ of *MSRB3* and all of *HMGA2*. However, despite the presence of strong associations with phenotype and a highly localized signal, the number and identity of variants in the region with functional effects that control body mass and ears is unclear. Dissection of the haplotype structure in this region is consistent with the hypothesis that two tightly linked loci control the ear type and body mass traits independently. A number of SNPs immediately downstream of *MSRB3* are good candidates for controlling ear type, whereas SNPs within the *HMGA2* gene are good candidates for influencing body mass.

The correlations between body mass and variation in the *HMGA2* locus are consistent between this study and another that assayed variation at this locus [21]. Interestingly, the best marker reported by ref. [21] (CFA10:11,335,165) was not identified in our study due to extremely low read depth in its vicinity. Conversely, all but two of our top nine highly associated body mass SNPs (Figure 3a) are not identified by ref. [21]. A cluster of three of the most associated SNPs that we identified upstream of *HMGA2* (CFA10:11,074,911; 11,075,912; 11,077,807) lie within an 18.0 kb interval where no SNPs were identified by ref. [21]. A second cluster of four highly associated SNPs that we identified within the *HMGA2* gene (CFA10:11,365,382; 11,365,899; 11,366,464; 11,366,743) lie with a 4.2 kb interval where no SNPs were found by ref. [21]. One of the SNPs shared by both studies (CFA10:11,162,577) shows a highly similar pattern of segregation to best SNP in ref. [21]. Out of the 15 small breeds that we inferred haplotypes for, 8 were also genotyped at the best marker reported by ref. [21]. The results were consistent with the derived variant at the best marker being found on the small haplotype reported here. Additional genotyping and functional studies will be necessary to determine which variants in the region directly influence body mass.

These two genes are both strong candidates for involvement in phenotypic variation. The *MSRB3* gene encodes zinc-containing methionine sulfoxide reductase B3, which catalyses the reduction of methionine sulfoxide to methionine [27]. It is involved in stress resistance and longevity in *Drosophila* [28] and has a antimicrobial effect [29]. A nonsynonymous mutation has been identified that causes deafness in a human family and the expression of *MSRB3* in the inner ear is essential for hearing [22]. It is however, unknown if it exerts an effect on outer ear morphology. GWAS have identified variants within *MSRB3* that are associated with the timing of primary tooth development during infancy [30, 31]. Tooth development is a highly heritable and interacts with the development of the entire craniofacial complex [30]. Differences in DNA methylation in the promoter region of *MSRB3* correlate with gestational age at birth [32]. The associations with craniofacial development and hearing therefore suggest *MSRB3* as a highly plausible candidate for influencing ear development.

The SNPs with highest association to ear type are found outside protein coding genes, and are not known to be within transcribed elements in dog. However, the orthologous region in the human genome contains several lincRNAs. Closer inspection of the cDNA evidence from which these lincRNA models were curated suggests expression in multiple tissues. If these lincRNAs are genuine transcripts in dog, it is possible that they could affect expression of one or both of the flanking genes, as has been described for other lincRNAs in human and mouse [33, 34]. A targeted analysis of expression during developmental stages relevant for ear development would be necessary to demonstrate their existence and potential connection with ear phenotype.

Several candidate variants for affecting body mass are found within an intron of *HMGA2* [35] which is implicated as a regulator of transcription and in the proliferation and differentiation of cells during development [36]. The expression of *HMGA2* in adult tissues is commonly associated with both malignant and benign tumour formation [37] and a role in adipogenesis and mesenchymal differentiation [37] has been suggested. Variants in *HMGA2* have been identified that are associated with height [24, 25], head circumference [38] intracranial volume [39] and permanent dentition [40]. This gene is also therefore a strong candidate for influencing both ear morphology and body mass. One of the SNPs associated with body mass is found within a conserved element within an intron of *HMGA2* that is likely to coincide with transcription factor binding activity that could affect expression of this gene.

We cannot fully rule out the possibility that a single locus with multiple alleles, such as a copy number variant, that we have been unable to detect controls both body mass and ear type. One drawback of assaying variation by using sequence capture and mapping of short reads to the reference genome is that we are unable to comprehensively assay copy number variation caused by insertion of sequence not present in the reference. It is therefore possible that structural variation involved in phenotypic variation is undetected.

Other loci elsewhere in the genome are also likely to influence ear type and body mass. In this study, ear type was considered a quantitative trait, and breeds that did not clearly have marked drop or prick type ears were classified as intermediate. It is possible that more detailed ear classification schemes could identify other loci that influence ear morphology using a GWAS approach. Furthermore, variants in at least six other loci associate with small size [21]. Here we find that the variants that associate with small size and drop ears in the CFA10 region very rarely occur on the same haplotype, and such recombinant haplotypes are not common in in the small, drop ear breeds in our dataset. This indicates the importance of other loci in determining both body mass and ear type in dog breeds.

We gained further insight into the evolutionary forces affecting the CFA10 region by analysing levels of heterozygosity and F_ST_ compared with wolves compared to the rest of the genome. In the region close to the drop ear associated SNPs there is very high heterozygosity in a heterogeneous sample of breeds, reflecting the presence of multiple SNPs with highly divergent frequencies between breeds. However, the regions immediately surrounding the associated interval exhibit extremely low levels of variation in dogs and high F_ST_, consistent with selection at domestication. We therefore hypothesize that these regions harbour one or more variants affected by selection related to dog domestication. This suggests that genetic variation in this region may have experienced two phases of selection: one related to dog domestication, which led to dog specific morphologies and/or behaviour, and another accompanying breed creation that led to differences in body mass and ear morphology between dog breeds. It is however, unlikely that the specific drop ear haplotype identified in this study was selected during early domestication, as it is associated with a relatively extreme phenotype that is exhibited only by a subset of breeds.

The findings presented here demonstrate how response to artificial selection in domestic species can be affected by the genetic architecture of the trait under selection. There are a large number of examples of traits that commonly appear together. For example, there is an association between white coats and deafness in both cats and dogs [16, 41]. Chickens with “frizzle feathers” that curl outward rather than lying flat against their bodies also have physiological abnormalities and lay fewer eggs compared to wild type [42]. Hair greying with age is associated with susceptibility to melanoma in the horse [43]. Such observations are generally expected to be due to pleiotropy at single loci and in some cases the identity of specific variants have been identified. For example mutations in α-keratin (*KRT75*) produce the pleiotropic effects in chicken [42] and a *cis*-acting regulatory mutation in intron 6 of *STX17* (syntaxin-17) is responsible for the pigmentation and melanoma susceptibility phenotypes in horse [43].

Drop ears are observed in several domestic animals including dogs, rabbits, cattle, pigs and goats and are often associated with other traits such as piebald coats, curly tails and smaller skulls. A similar suite of traits has also been observed in foxes selected for tameness in the classic Farm-Fox experiment [19]. The leading explanation for these associations between traits is that they are due to pleiotropy, which implies that genetic variants affecting behaviour also have effects on morphology. However, in some cases, such associations between different traits can be produced by genetic linkage between more than one variant that govern them separately. This study has revealed an example of this case, where variants governing body mass and ear type are in genetic linkage.

## Conclusions

We have characterised a region on canine chromosome 10 that contains genetic variants that affect ear type and body mass. We suggest candidate mutations for both of these traits and provide further evidence that the region may have been under selection during dog domestication. This study demonstrates how the presence of linked variants influencing different traits could limit the combinations of phenotypes available for selection. Such genetic correlations restrict the palette available for both natural and artificial selection to work with to produce desired phenotypes and can reduce the rate of genetic adaptation.

## Methods

### Ethics statement

All DNA samples were collected from privately owned pet dogs with the owners’ consent according to relevant national and international guidelines. Ethical approval was granted by the Ethical board for experimental animals in Uppsala, Sweden (Dnr C138/6) and from US dogs by the Broad Institute based on a protocol approved by the MIT CAC (*0907-068-10 and 1109-127-12).

### GWAS

Genome wide association studies for variation in body mass and ear morphology were performed using a dataset of 509 dogs from 46 breeds reported in Vaysse *et al.* [12] (See Table 1). Ear types were encoded as drop (14 breeds), prick (12 breeds) or intermediate (20 breeds) based on standard breed descriptions. Breeds with an ear type that hangs by the side of the head were classified as drop ear such as Beagles, Spaniels, Setters and Weimaraner. Breeds were ears stand erect such as German Shepherds, Chihuahuas, Elkhounds and Spitz breeds were classified as prick ear breeds. Breeds that did not clearly fit into those categories were classified as intermediate. These include breeds with cocked ears such as Collies and the partially erect button ears found on many Terriers. Photographs of representative breeds are shown in Additional file 12: Figure S5. GWAS were performed using a quantitative association coding the three ear type classifications as prick = 1, intermediate = 2 and drop = 3. We next performed a quantitative association with body mass using all 46 breeds, using the breed average values for sex-averaged mass in kg (Table 1). We used a breed permutation procedure to estimate significance of associations [12]. This involved permuting the breed-averaged trait values among breeds, always assigning an identical phenotype to every sample from the same breed. Genomewide significance level (EMP2) was estimated by comparing observed chi-squared values at each SNP with the maximum chi-squared value observed at any SNP in the genome in breed-permuted datasets. GWAS were performed using plink [44] and a custom perl script. We also performed quantitative GWAS for body mass within each of the three ear categories using the same procedure. We tested for correlations between ear type and body mass using a Kruskal–Wallis one-way analysis of variance using ear type as a factor.

### Resequencing of a 3 Mb region

We performed sequence capture (SC) of 5 libraries, each comprised of a pool of 5 samples from a single dog breed, to enrich for a 3 Mb region 9.5–12.5 Mb on CFA10 (canFam2.0). The breeds used included two small breeds with non-drop ears (Border Terrier, Jack Russell Terrier) one large breed with non-drop ears (German Shepherd) and two large breeds with drop ears (Weimaraner, English Springer Spaniel) (Table 2). The breeds were chosen to be different from those used in Vaysse *et al.* [12]. The sequence capture was performed using a Roche NimbleGen array containing probes designed to hybridize to the region. 96.1 % of the target had a probe within a distance of 100 bp.

This was followed by sequencing of each library on a single lane of Illumina Hi-Seq to produce 100 bp paired end reads, leading to increased mapping accuracy compared to a previous study using single reads [12]. The short reads were aligned to the complete CanFam2.0 reference genome using BWA [45], followed by sorting and indexing of bam files and addition of read groups using picard [46]. Additional quality control steps including realigning around indels and removal of PCR duplicates were performed using GATK [47]. All samples from the SC dataset had coverage of >98 % of the 3 MB region with an average of 73.4 % of bases >100x. There are no large assembly gaps in the region. The average insert size of the paired-end library was 256 bp.

We used the alignments resulting from the quality control steps to produce pileup files using samtools [48], from which we called SNPs using a custom algorithm. We first scanned all pileup files and filtered out sites with <100x coverage as this represents only ~2 % of the average coverage across the whole region. At each site, bases with phred quality <20 were not considered. We then compared base counts across all five pools at each position in the region. Only sites with >10 % of reads mapping to a minor allele were considered in total across all pools were considered as variable SNPs for further analysis. The pattern of segregation at each SNP in each pool was classified as fixed reference, fixed non-reference, heterozygous or missing if the site had low coverage (see above). We considered different thresholds for fixation, a very loose cutoff where 70 % of reads needed to match the same allele for it to be considered fixed in a pool, a loose cutoff where 90 % of reads needed to support the same allele and a stricter one where 99 % needed to support the same allele. SNPs in pools where no allele was found at a higher frequency than these cutoffs were considered to be polymorphic in that pool.

We noted read coverage was lower and more variable in the English Springer Spaniel pool (ESS) compared with other pools. Sites from this breed were therefore considered missing unless a call could also be made from the other breed with the same phenotype (Weimaraner; WEI) thus preventing the pattern of segregation in breeds with this phenotype from only being represented by the ESS sample. In order to be a considered a candidate variant for affecting body mass, a SNP was required to be fixed for one allele in the two pools derived from small breeds and fixed for another allele in all the three pools derived from the large breeds, whereas to be considered a candidate for affecting ear type, it was necessary that one allele was fixed in the two pools from drop ear breeds and another allele fixed in the three pools from prick ear breeds. The patterns of segregation among breeds of each phenotype were used to identify candidate mutations for the ear and body mass phenotypes using both loose and strict criteria.

### Comparison with pooled whole-genome resequencing data

We compared variants identified in SC data to the sequence data in the same region from whole genome sequencing (WGS) of pools of samples used in the Axelsson *et al.* study [10]. Out of five dog pools in this study, none contained small breeds, two (pool 3 and pool 4) consisted only of breeds with drop ears, one (pool 5) consisted of a single prick eared breed, and two consisted of breeds with a mixture of ear types (pool2 and pool 6; Table 2). The wolf pool (pool 1) was included in the analysis as large and prick eared. The SNP positions identified in SC data were compared to the nucleotides aligned in pileups of each pool and examined for differences in allele frequencies. SNPs in each pool were considered to be consistent as a candidate for ear type or body mass if they were inferred as fixed for the same allele, inconclusive if a confident call could not be made, and inconsistent if the alternate allele was fixed. Because of the much lower coverage of these pools, a SNP was only considered as fixed for a particular allele if it was supported by all of the reads.

Candidate SNPs were selected as those with the strongest correlation between patterns of segregation and either the drop or prick ear trait. This was performed by taking the SNPs that were selected as candidates for association with a trait in the SC pools, and comparing patterns of segregation in the WGS pools. Candidates from the SC pools were not considered further if one or more of the WGS pools were fixed for an allele inconsistent with an association with phenotype. We gave highest priority to candidates that matched according to the strict cutoff for fixation where 99 % of reads were required to match an allele for it to be considered fixed in a pool. Additional candidates were taken from the looser cutoff definitions. Within the critical interval 11.0–11.5 Mb, only 12 SNPs were identified with a cutoff of 70 %, of which all were included in the subsequent SNP panel. Within the same interval, 80 body-mass-associated SNPs were included in the SNP panel out of 145 identified at the 70 % cutoff but only 83 identified at the 90 % cutoff.

We also utilised the combined SC and WGS pooled resequencing data to identify SNPs fixed for alternate alleles in all sequenced dogs compared to the wolf pool. These were identified as those with more than 3000x coverage in total in all dog pools and >3x coverage in the wolf pool and fixed for alternative alleles, with no more than 1 % of reads supporting a different allele. This produced a list of potential functional variants involved in selection related to dog domestication.

### CNV analysis

We analysed the SC reads to identify potential structural variants that could explain the difference in phenotypes coverage by looking for variation in coverage across the 3 Mb region. This was done by scanning each pileup file in nonoverlapping sliding windows of 100 bp. We first calculated the depth of coverage in each window relative to the average coverage in the pool across all windows. These values were then normalised across pools to identify deviations in relative coverage in subset of pools in specific windows, which could result from the presence of copy number variation in a particular region.

We scanned the critical region 11.0–11.5 Mb on CFA10 for windows with asymmetrical depth of coverage across pools, focussing on windows with coverage where one or more breeds had coverage more than two times the average or where one or more breeds completely lacked coverage. The distributions of reads in these windows were then manually inspected in Integrative Genome Viewer (IGV) [49] and cross referenced with genes, conservation and repetitive elements.

### SNP genotyping and analysis

Candidate SNPs for controlling variation in ear type and body mass were first selected using the strict criteria for fixation in a pool and supplemented with SNPs identified using loose criteria (see above). We selected a panel of 123 SNPs for genotyping in a larger sample of breeds from the candidate SNPs identified in the sequencing data. We genotyped these SNPs in 288 samples from 46 breeds selected for wide variation in both body mass and ear type (Table 3) using the Illumina Golden Gate assay with standard protocol. We identified SNPs with the strongest association to the body mass and ear phenotypes by performing association studies using point-wise breed permutations for each SNP to determine significance, comparing the true chi-squared value at each SNP with chi-squared values of at that SNP from permuted datasets. We regarded ear type as a quantitative trait with three values: drop ear, intermediate ear and prick ear. We included two pairs of breeds that are closely related to each other but differ to some extent in ear type. These are Norwich Terrier (prick ear)/Norfolk Terrier (drop ears) and the Papillon (prick ear)/Phalène (drop ear). Compared to all other breeds, these breeds are classified as intermediate ear.

We selected 15 SNPs with the strongest associations to either ear (*p* < 10^−45^) or body mass phenotypes (*p* < 10^−15^) and inferred haplotype patterns across these SNPs using the EM algorithm implemented in plink, assigning the most probable pair of haplotypes to each sample. We then enumerated the set of haplotypes associated with each of the breed phenotypes.

### Selective sweep analysis

We analysed F_ST_ and heterozygosity across the whole genome using the data from Axelsson *et al.* [10]. This was done by dividing the genome into 40 kb windows. We measured heterozygosity across all dog WGS pools and F_ST_ between the dog and wolf WGS pools across the genome as described in ref. [10].

### Functional candidates

We identified annotated elements that associate with candidate functional mutations by cross-referencing with both the dog and human genome annotations as well as conserved elements (GERP, [50]) and conserved elements from 100 vertebrates (EnsEMBL 74, [51]). In order to transfer human annotations, we first generated a synteny map between the dog genome (canFam2.0, EnsEMBL release 64) and human (release 37, EnsEMBL release 74) using the Satsuma genome aligner [52]. Next, we employed the genomic coordinate translator Kraken [53]. Kraken first identifies candidate regions for projection from the synteny graph and then performs an exhaustive alignment to return base-accurate lift-over annotations. Each SNP was manually evaluated for overlap with annotated or projected features.

We also translated the dog coordinates onto the human genome build and analysed predicted effects using the Variant Effect Predictor (http://www.ensembl.org/info/docs/tools/vep/index.html).

## Data availability

All sequence data has been submitted to NCBI Sequence Read Achive under BioProject ID PRJNA253907.

## Additional files

Additional file 1: Table S1. Result of GWAS studies for ear type, body mass, and body mass controlling for ear type (drop, prick and intermediate). Both raw *p*-values and *p*-values from breed permutation procedure are shown for each analysis. EMP1 is the point-wise breed-permuted *p*-value derived from comparison of the observed significance value at each SNP with the significance values of 1000 permutations at the same SNP. EMP2 is the genome-wide breed-permuted *p*-value derived from comparison of the significance value at each SNP with the maximum significance values of 1000 permutations of all SNPs in the genome. SNPs with genome-wide *p*-values < 0.05 are highlighted. Only SNPs with EMP1 < 0.05 are shown. Additional file 2: Figure S1. GWAS of body mass within classes defined by ear type. a) prick ear breeds (12 breeds; average body mass 24.6 kg), b) intermediate ear breeds (18 breeds; average body mass 24.2 kg), c) drop ear breeds (16 breeds; average body mass 22.9 kg). Raw *p*-values are shown and point-wise genome wide significance by breed permutation (1000 permutations) are shown for most significant SNPs. Prick ear breeds show body mass association with the *IGF1* region on CFA15, drop ear breeds do not show any significant associations with body mass. For intermediate ear breeds, the strongest association is shown in the CFA10 region also associated with ear type. Additional file 3: Table S2. Patterns of segregation and SNP genotype calls from resequencing data in 11 pools of dog samples. Allele counts in each pool from Table 2 are shown. Candidate SNPs for each trait from the SC pools and concordance with the WGS pools are marked. Correspondence with conservation tracks annotated to the dog canFam2.0 genome sequence and human genes mapped to this sequence are shown. SNPs selected for further genotyping are also marked. Additional file 4: Figure S2. Relative variation in read depth in 600 kb encompassing the critical interval in four breeds (Weimeraner, WEI; Border Terrier, BT; German Shepherd, GS; Jack Russell Terrier, JR). Read depth is measured in 100 bp windows and normalized across breeds and across the region. Additional file 5: Table S3. Regions identified with variable depth of coverage between pools of dog breeds from the SC data that could potentially represent copy number variation. Relative depth of coverage in four dog pools from the SC data are shown along with the correspondence with repeat elements, conservation and gene tracks mapped on the canFam2.0 reference genome. Additional file 6: Table S4. Matrix of SNP genotypes of 288 samples from 46 dog breeds typed at 123 SNPs. Phenotypes of breeds in terms of ear type and body mass are shown, along with strength of associations between phenotype and genotype. SNP alleles are shown in the top row, with the major allele denoted as “a” and the minor allele as “b”. Additional file 7: Table S5. Correlation between body mass and ear traits in SNP genotyping dataset. Both raw *p*-values and point-wise permutation values (EMP1) are shown, along with annotations from conservation and gene annotation tracks relative to canFam2.0. SNPs with strongest associations to ear type (*p* < 10^−45^) or body mass (*p* < 10^−15^) are highlighted. These SNPs were included in haplotype inference. Additional file 8: Figure S3. Association between genetic variation and body mass at 123 SNPs in subsets of breeds divided according to ear type. Additional file 9: Table S6. Haplotypes inferred for all genotyped samples using the E-M algorithm. a) The most probable pair of haplotypes for each sample. b) Haplotype counts for each breed, including the sum of breeds with similar ear and body mass phenotypes. c) Identity and total haplotype counts in the entire dataset. Additional file 10: Figure S4. Pairwise linkage disequilibrium between the 15 SNPs used to infer haplotype structure measured using r^2^. Additional file 11: Table S7. List of SNPs inferred to be fixed for alternative alleles between dogs and wolves. Number of reads matching each allele in dog and wolf are shown. Conservation track from canFam2.0 is also shown. Additional file 12: Figure S5. Photographs of breeds representative of the three ear categories used in this study. a) Prick ear breeds: German Shepherd, Chihuahua, Schipperke. b) Intermediate ear breeds: Jack Russell Terrier, Schnauzer, Border Collie. c) Drop ear breeds: Irish Setter, English Springer Spaniel, Weimaraner. Attribution (in order of appearance): Marilyn Peddle, Howard Walfish, Thomas & Dianne Jones, Wikimedia Commons user Sellys, Flickr user SheltieBoy, Wikimedia Commons user Lilly M, Flickr user timricketts62, Steven Lilley, Monique Gidding.

## Abbreviations

LD: Linkage disequilibrium
SNP: Single nucleotide polymorphism
GWAS: Genome wide association study, SC, Sequence capture
WGS: Whole genome sequencing
CNV: Copy number variant
IGV: Integrative genome viewer
ChIP-seq: Chromatin immunoprecipitation sequencing
GATK: Genome analysis toolkit
