# Supplementary figures and images for: Linked genetic variants on chromosome 10 control ear morphology and body mass among dog breeds
Source: BMC Genomics. 2015 Jun 23;16(1):474. doi: 10.1186/s12864-015-1702-2 (PMC4477608; doi:10.1186/s12864-015-1702-2)

a)

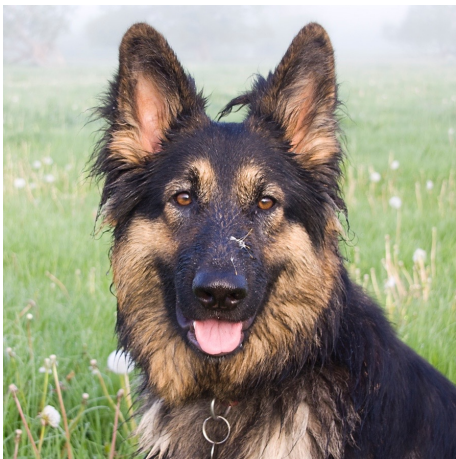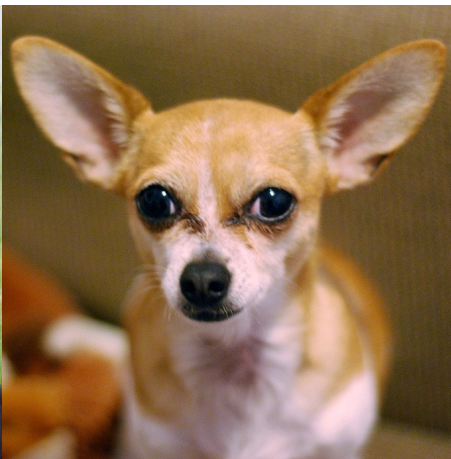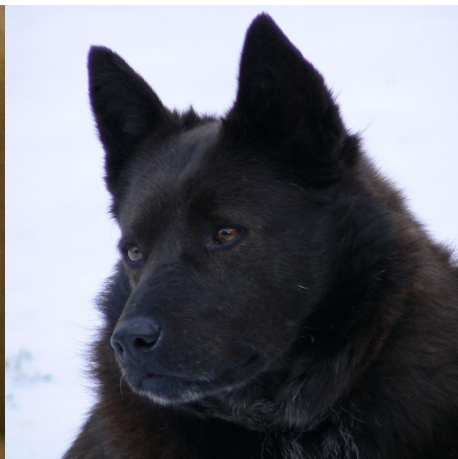

b)

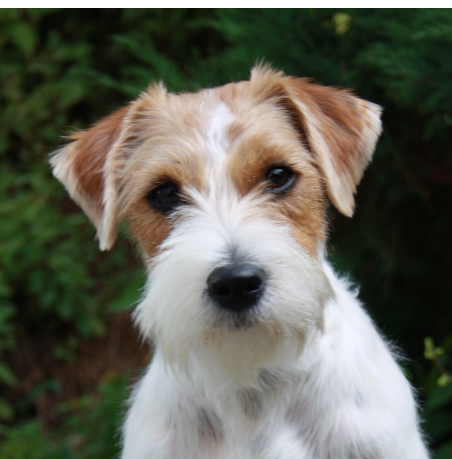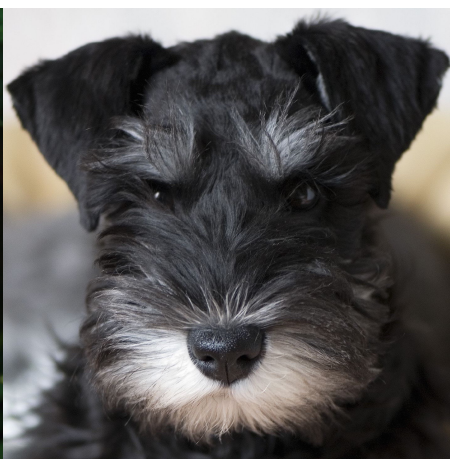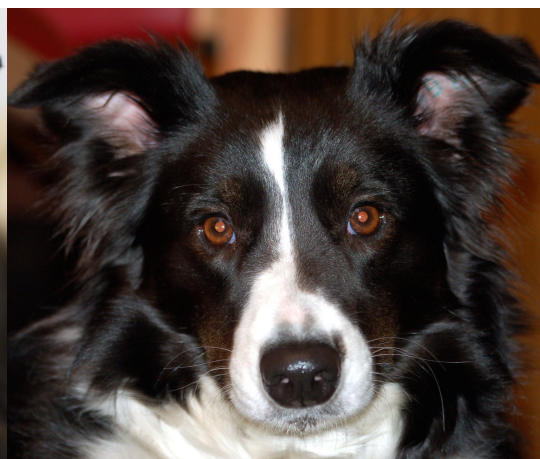

c)

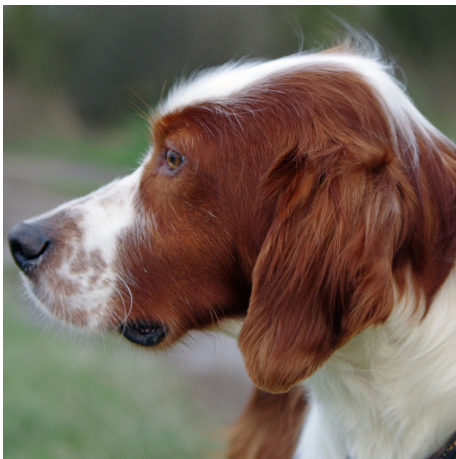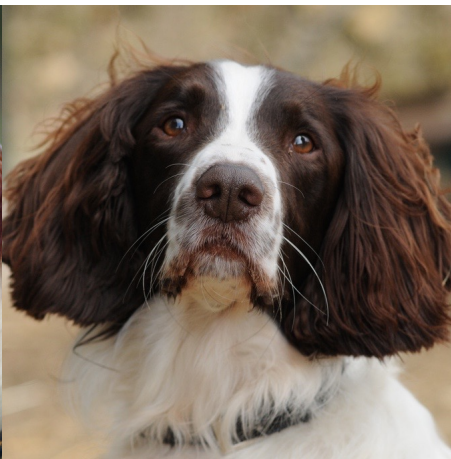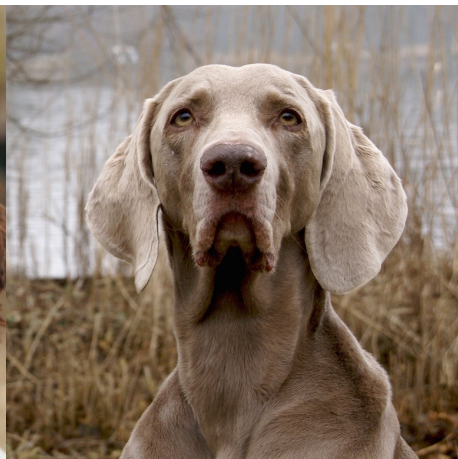

Supplement: Additional file 12: Figure S5. — Photographs of breeds representative of the three ear categories used in this study. a) Prick ear breeds: German Shepherd, Chihuahua, Schipperke. b) Intermediate ear breeds: Jack Russell Terrier, Schnauzer, Border Collie. c) Drop ear breeds: Irish Setter, English Springer Spaniel, Weimaraner. Attribution (in order of appearance): Marilyn Peddle, Howard Walfish, Thomas & Dianne Jones, Wikimedia Commons user Sellys, Flickr user SheltieBoy, Wikimedia Commons user Lilly M, Flickr user timricketts62, Steven Lilley, Monique Gidding. [file 12864_2015_1702_MOESM12_ESM.pdf]
